# Recombinant humanized type VII collagen for skin repair and regeneration: prospects for reconstructing the dermal-epidermal junction

**DOI:** 10.3389/fmed.2026.1912442

**Published:** 2026-08-12

**Authors:** Yanyan Lin, Jie Zheng, Xiaobin Lan

**Affiliations:** 1 School of Industry, Shanxi Medical University, Jinzhong, Shanxi, China; 2 School of Nursing, Shanxi Medical University, Jinzhong, Shanxi, China; 3 Shanxi Key Laboratory of Functional Proteins, Shanxi Jinbo Bio-Pharmaceutical Co., Ltd., Taiyuan, Shanxi, China

**Keywords:** anchoring fibrils, dermal-epidermal junction, recombinant humanized type VII collagen, skin regeneration, type VII collagen, wound healing

## Abstract

Type VII collagen (Col VII) is a key component of the anchoring fibrils at the dermal-epidermal junction (DEJ). It plays a crucial role in maintaining stable adhesion between the epidermis and dermis, promoting wound re-epithelialization, and ensuring the structural integrity of the skin following repair. With advancements in genetic engineering, synthetic biology, and recombinant protein expression technologies, recombinant humanized type VII collagen (rhCol VII) has gradually emerged as a novel functional biomaterial of interest in the fields of skin repair and regenerative medicine. Compared with traditional type I and type III collagen materials, the potential advantages of rhCol VII lie in its excellent safety profile, scalability for industrial production, and its potential structural and functional targeting of the DEJ, which may offer new insights into restoring interface stability following wound repair. This review summarizes the molecular structure, biological functions, and recent therapeutic research progress of Col VII, as well as the application prospects of rhCol VII in chronic wounds, post-aesthetic surgery barrier damage, and tissue-engineered skin. It also discusses the challenges that remain to be addressed in material development and clinical translation, with the aim of providing a reference for further research on rhCol VII-based skin repair materials.

## 1 Introduction

The skin is an important barrier organ of the human body, playing a key role in defending against external stimuli and maintaining internal stability (1, 2). Factors such as trauma, inflammation, photoaging, and aesthetic procedures can all damage the skin barrier and impair tissue repair (3, 4). Skin wound healing is a continuous and complex process that involves not only wound closure but also re-epithelialization, extracellular matrix remodeling, and the restoration of tissue stability (5, 6). An imbalance in this healing process can lead to delayed wound healing, chronic inflammation, or scar formation (7–9). Therefore, improving the structural integrity and functional stability of repaired skin has become a critical issue in the fields of skin repair and regenerative medicine.

Collagen-based materials have been widely used in wound healing and regenerative medicine research due to their excellent biocompatibility, ability to support cell adhesion, and value as tissue engineering scaffolds (10, 11). Previous studies have primarily focused on type I and type III collagen, both of which play crucial roles in dermal matrix formation, maintenance of tissue mechanics, and wound remodeling (12, 13). However, the key to skin repair is not limited to the regeneration of the dermal matrix. The DEJ is the critical interface connecting the epidermis and dermis, and its structural integrity determines whether the newly formed epidermis can stably adhere to the underlying dermal matrix (14). Against this backdrop, DEJ repair centered on collagen VII has gradually emerged as a crucial entry point for understanding high-quality skin repair.

In recent years, research and industrialization of recombinant collagen materials have shifted from extraction from animal tissues to human-derived sequence design, heterologous expression, and large-scale production, gradually becoming a major focus in the study of skin repair materials (15, 16). Compared to animal-derived collagen, recombinant humanized collagen offers advantages such as a clear source, controllable quality, and lower risks associated with animal-derived materials (17, 18). rhCol VII is a recombinant protein designed based on the structural and functional characteristics of human Col VII. Compared to traditional collagen materials, rhCol VII enhances the structural specificity, controllability, and translational potential of the material through humanized design (19, 20). This review focuses on rhCol VII, summarizing its structural basis, expression and function, application scenarios, and translational challenges, with the aim of providing a reference for further research on rhCol VII in skin repair materials and regenerative medicine.

## 2 Structural and biological basis of Col VII

### 2.1 Structure and distribution of Col VII

Col VII is an extracellular matrix protein encoded by the human *COL7A1* gene on chromosome 3p21 (21, 22). It is the core component of anchoring fibrils in the DEJ and is central to stable adhesion between the epidermis and the dermis. Structurally, Col VII is a large homotrimeric collagen composed of three identical α1(VII) chains (23). Each α1(VII) chain contains a central collagenous triple helix flanked by non-collagenous domains. The central triple-helical domain is characterized by repeating Gly-Xaa-Yaa sequences, which form the basic molecular scaffold and tensile framework of Col VII (22). The flanking domains include a small C-terminal non-collagenous 2 domain (NC2) and a larger N-terminal non-collagenous 1 domain (NC1) (24).

The NC1 domain contains multiple subdomains involved in extracellular matrix adhesion. It mediates interactions between Col VII and laminin-332, type IV collagen, and other basement membrane components (25, 26). These interactions provide a structural basis for Col VII localization to the DEJ and for its role in bridging the basement membrane zone. The NC2 domain mainly participates in antiparallel dimerization between Col VII molecules and supports subsequent anchoring fibril assembly (27). Thus, Col VII function depends not only on the collagen triple helix but also on NC1- and NC2-mediated matrix interactions and molecular assembly.

### 2.2 Assembly of Col VII

**Figure 1:**
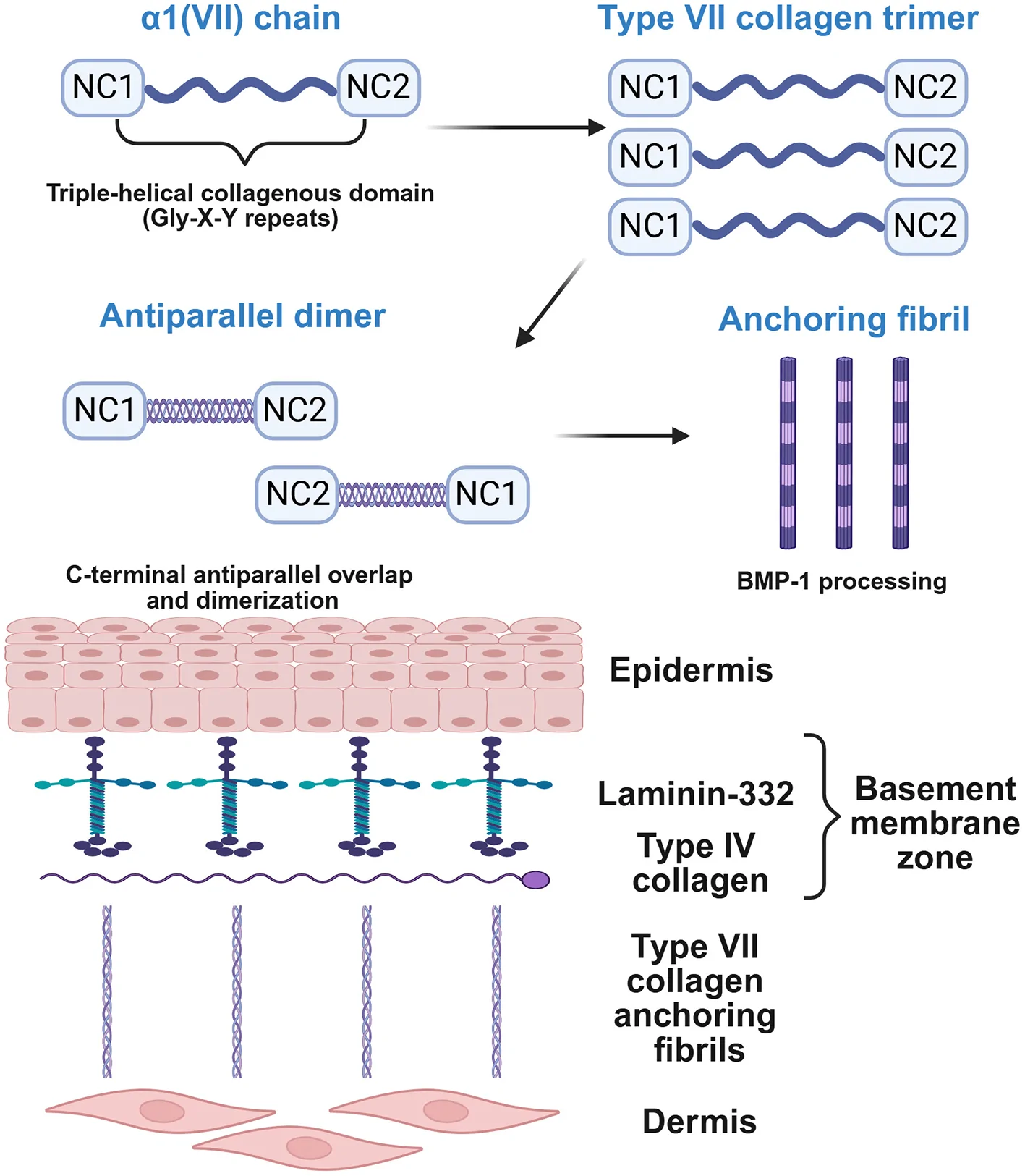
*Structural organization, assembly, and DEJ localization of Col VII*. Col VII is composed of an NC1 domain, a central triple-helical collagenous domain, and an NC2 domain. Three α1(VII) chains assemble into a Col VII trimer, followed by NC2-mediated antiparallel dimerization and proteolytic processing to form anchoring fibrils. At the DEJ, Col VII anchoring fibrils connect basement membrane components, including laminin-332 and type IV collagen, with the underlying dermal matrix. Created with BioRender.com.

Col VII is synthesized mainly by epidermal keratinocytes and dermal fibroblasts and is deposited primarily in the DEJ (28). Its function depends on both protein expression and correct extracellular assembly. After secretion, procollagen VII forms a homotrimer and two molecules undergo antiparallel dimerization through the C-terminal NC2 domain (27). In this process, the NC2 domain of one procollagen VII molecule recognizes and binds the triple-helical region adjacent to Cys-2625 in another procollagen VII molecule (29). The resulting dimers are stabilized by disulfide bonds between cysteine residues in the NC2 and triple-helical domains. After proteolytic processing by bone morphogenetic protein-1 and related proteinases, mature anchoring fibrils gradually form (30). Mature Col VII binds laminin-332, type IV collagen, and other basement membrane components through NC1 and links them to the dermal collagen network below. Through this arrangement, the epidermal basement membrane and dermis become a mechanically continuous structural unit (26). The structural organization, supramolecular assembly, and DEJ localization of Col VII are summarized in Figure 1.

### 2.3 Biological roles of Col VII

#### 2.3.1 Maintaining the stability of the DEJ

Col VII is the major structural component of anchoring fibrils and can assemble into anchoring fibrils that connect the epidermal basement membrane with the underlying dermal matrix. This connection can help the skin resist external mechanical stress and provide the structural basis for stable dermal–epidermal adhesion (31). Mutations in *COL7A1* or reduced Col VII expression may compromise DEJ stability, making the skin more susceptible to tissue cleavage and blister formation after minor friction or traction.

#### 2.3.2 Contributing to wound repair and DEJ reconstruction

After wounding, the original DEJ and basement membrane zone are disrupted. Newly formed epidermis must not only cover the wound surface but also re-establish stable attachment to the underlying dermal matrix (32). In this process, Col VII deposition at the newly formed DEJ may contribute to dermal–epidermal reconnection and provide structural support for basement membrane zone reconstruction and post-repair interface stability. Meanwhile, Col VII may also help maintain the organized distribution of basement membrane-associated adhesion molecules and enhance stable adhesion between basal keratinocytes and the basement membrane, thereby creating favorable conditions for keratinocyte migration, re-epithelialization, and continuous reconstruction of the newly formed DEJ (33).

#### 2.3.3 Potentially affecting the wound repair microenvironment

As wound repair progresses from epidermal coverage to granulation tissue formation and tissue remodeling, the role of Col VII may further extend to the local repair microenvironment. In *COL7A1*-deficient settings, granulation tissue formation and maturation may be altered, and wound repair is often accompanied by dysregulated fibroblast migration, cytokine expression, and extracellular matrix remodeling (33). These changes may disrupt the orderly transition from inflammation to tissue remodeling and reduce the quality of repair. Given that TGF-β signaling is closely associated with inflammatory cell recruitment, fibroblast activation, collagen deposition, and fibrotic responses, Col VII abnormalities caused by *COL7A1* deficiency may indirectly participate in the regulation of the wound repair microenvironment and repair quality by affecting fibroblast behavior and cytokine networks (34).

### 2.4 Biosynthesis and source limitations of Col VII

Traditional collagen materials are primarily derived from animal tissues, with common sources including cattle, pigs, and fish (35). In contrast, Col VII is not a major collagen type widely distributed in ordinary connective tissue; rather, it is primarily localized at the DEJ of the skin and certain mucous membranes. Due to its limited tissue distribution, high molecular weight, and complex domain structure, traditional animal tissue extraction methods struggle to yield stable, scalable, and structurally well-defined Col VII raw materials (31). Consequently, the development of Col VII materials cannot simply follow the traditional extraction and purification routes used for animal-derived collagen; instead, it relies more heavily on technical approaches such as recombinant expression, humanized sequence design, and functional fragment optimization.

## 3 Therapeutic and repair-related research on Col VII

### 3.1 DEB-related therapeutic research

Dystrophic epidermolysis bullosa (DEB) is currently the disease area where research on Col VII restoration therapies is most concentrated. Its core pathological basis involves abnormalities in the *COL7A1* gene leading to the absence or functional impairment of Col VII, which in turn results in insufficient formation of anchoring fibrils and instability of the DEJ. Given its well-defined etiology and clear therapeutic targets, DEB provides a relatively well-defined disease model for validating Col VII restoration strategies (36). Current Col VII-related therapeutic research mainly encompasses *COL7A1* gene therapy, recombinant Col VII protein replacement, cell-based therapy, RNA-based approaches, and genome editing.

#### 3.1.1 COL7A1 gene therapy

Beremagene geperpavec (B-VEC) is a topical gene therapy based on a replication-defective herpes simplex virus type 1 (HSV-1) vector that delivers *COL7A1* to wound-resident keratinocytes and fibroblasts, thereby restoring local Col VII expression and anchoring fibril formation (37). It can be applied directly to accessible wounds, repeated as needed, and does not require skin biopsy, individualized cell expansion, surgical grafting, or systemic conditioning. Currently, Phase I/II studies showed favorable local tolerability and wound-healing activity (38), and a phase III randomized controlled trial further showed that B-VEC-treated wounds achieved higher complete closure than placebo-treated wounds (39). Moreover, an open-label extension study also supported the long-term safety and tolerability of repeated B-VEC administration, providing further evidence for its continued use in the management of DEB wounds (40). Therefore, B-VEC is particularly advantageous for recurrent, superficial, and multiple accessible wounds that require repeated local management. However, B-VEC remains a topical, wound-directed therapy that requires repeated administration. Although longer-term extension data are encouraging, further follow-up may help clarify the durability of wound closure and long-term safety.

In addition to topical gene delivery, *ex vivo COL7A1* gene-modified cell therapy has also advanced clinically. In 2025, the US Food and Drug Administration approved prademagene zamikeracel (pz-cel; Zevaskyn), an autologous cellular sheet therapy based on *COL7A1* gene-modified cells, for the treatment of wounds in patients with recessive DEB (41). In this approach, patient-derived keratinocytes are genetically modified *ex vivo* to express functional Col VII, expanded into cellular sheets, and surgically applied to wounds. Compared with B-VEC, pz-cel combines molecular correction with epithelial replacement, making it more suitable for selected large, chronic, or refractory wounds that require structural coverage. Clinical studies of pz-cel reported improvements in wound healing, with no serious treatment-related adverse events observed (42). However, the clinical implementation of pz-cel remains relatively complex, as it requires patient biopsy, individualized manufacturing, specialized treatment centers, surgical wound-bed preparation, graft fixation, and postoperative protection. This procedure may be less suitable for patients with numerous small or frequently recurring wounds, and graft performance is closely influenced by wound-bed quality and postoperative immobilization. In addition, because pz-cel involves retroviral vector-mediated gene modification, potential insertional oncogenesis and the need for long-term follow-up should be considered, although such events have not been identified in available clinical studies.

#### 3.1.2 Recombinant Col VII protein replacement

Unlike *COL7A1* gene delivery, recombinant Col VII protein replacement is designed to provide the missing effector protein directly to the wound or DEJ, rather than inducing cells to produce Col VII through genetic correction. Woodley et al. showed that intradermal injection of rhCol VII restored Col VII function in DEB-related skin models (43). Remington et al. further demonstrated in *COL7A1*-deficient mice that locally injected rhCol VII could integrate stably into the DEJ, form anchoring fibrils, reduce skin fragility, decrease new blister formation, and extend survival; importantly, Col VII was detectable at the basement membrane zone as early as 6 h after injection, supporting the rationale for protein replacement as a relatively rapid structural intervention (44). Recombinant Col VII delivered topically has also been reported to promote re-epithelialization and wound closure, suggesting its potential application in recessive dystrophic epidermolysis bullosa (RDEB) wounds and other chronic cutaneous injuries (19).

Compared with B-VEC, which relies on repeated topical delivery of *COL7A1* to promote local Col VII expression, recombinant Col VII directly supplies the functional protein and may therefore provide more rapid structural support at the DEJ. Its local dose could also be adjusted according to wound characteristics and treatment response, supporting its potential use as an immediate, controllable intervention for acute or newly formed wounds. From a safety perspective, recombinant Col VII protein replacement avoids viral-vector administration and does not involve permanent genetic modification, which may reduce concerns associated with vector exposure and genomic alteration. Because the functional protein is delivered directly, recombinant Col VII may also become structurally available at the DEJ more rapidly than gene therapy, which requires cellular transduction followed by *COL7A1* expression and protein assembly. In addition, its local dose could potentially be adjusted according to wound size, depth, and repair response, supporting its use as an immediate and controllable intervention for acute or newly formed wounds. As a standardized, off-the-shelf local therapy, recombinant Col VII may also offer practical and potential cost-related advantages over individualized cell-based treatments, although direct pharmacoeconomic comparisons with B-VEC are currently lacking. These advantages should nevertheless be balanced against the complexity of producing structurally correct Col VII, its finite tissue persistence, the possible need for repeated administration, and the need to further evaluate immunogenicity and long-term safety. Overall, recombinant Col VII may occupy a distinct therapeutic niche as a rapid, dose-adjustable strategy for localized structural repair, complementing the longer-term molecular correction provided by B-VEC.

#### 3.1.3 Cell-based therapy

Cell-based approaches related to Col VII generally rely on donor cells to secrete Col VII after local administration or systemic homing, or to modulate the wound microenvironment through paracrine effects that improve DEJ stability (45, 46). In a mouse model of RDEB, intradermal injection of wild-type fibroblasts was reported to increase Col VII deposition at the DEJ. Although the injected cells appeared to survive only transiently, Col VII deposition remained detectable at the DEJ, suggesting that donor-cell-derived Col VII may retain functional relevance after cell loss (45, 47). Clinical studies have also reported that intradermal injection of allogeneic fibroblasts may increase local Col VII deposition and anchoring fibril formation with an acceptable safety profile. However, subsequent randomized studies suggested that clinical improvement was not consistently associated with increased Col VII deposition. These findings indicate that fibroblast-based therapy may involve both transient Col VII supply and paracrine support of local repair responses, rather than acting solely as a direct source of Col VII (46, 48).

Beyond fibroblasts, bone marrow-derived mesenchymal stromal cells (49), ABCB5-positive mesenchymal stromal cells, and hematopoietic stem cell transplantation have also been explored for RDEB treatment (50–52). These approaches are less directly controllable than recombinant Col VII protein replacement in terms of dose and local Col VII availability, but they may provide broader biological support through secreted factors, immune modulation, and interaction with the wound microenvironment. Compared with gene-corrected strategies, unmodified cell therapies may avoid direct genetic manipulation, but they do not provide durable molecular correction of *COL7A1*. Therefore, Col VII-related cell-based therapy may be better positioned as a complementary approach for improving wound repair quality, while limited donor-cell persistence, variable wound conditions, and less predictable therapeutic effects remain important challenges (53–56).

#### 3.1.4 RNA-based approaches and genome editing

RNA-based approaches and genome editing also offer new avenues for the precise correction of *COL7A1* mutations. RNA trans-splicing and antisense oligonucleotide-mediated exon skipping can correct abnormal *COL7A1* transcripts at the post-transcriptional level; studies have already observed the restoration of Col VII expression and the formation of anchoring fibril-like structures in patient-derived cells or skin-like models (57). Compared with permanent DNA editing, RNA-based strategies are potentially more reversible and may have a more controllable safety profile, but their therapeutic effect is generally transient and highly dependent on mutation location, splicing outcome, delivery efficiency, and repeated administration. Gene editing technologies, on the other hand, aim to correct pathogenic *COL7A1* mutations at the DNA level. Methods such as CRISPR/Cas9, base editing, and prime editing have demonstrated the potential to restore Col VII expression in preclinical studies (58, 59).

Genome editing strategies may provide a more durable form of molecular correction than RNA-based approaches, as they act at the DNA level. However, their translational feasibility remains closely linked to editing precision, delivery efficiency, off-target risk, genotoxicity assessment, and long-term monitoring. In this context, RNA therapeutics and genome editing represent two complementary precision strategies: RNA-based approaches may be more suitable for reversible and mutation-specific transcript modulation, whereas genome editing may be more relevant to durable correction but requires more extensive preclinical and clinical safety evaluation before broader application.

### 3.2 Col VII autoimmunity in EBA

Epidermolysis bullosa acquisita (EBA) is an acquired autoimmune subepidermal blistering disease mediated by autoantibodies against Col VII, the major component of anchoring fibrils at the basement membrane zone (60, 61). In contrast to DEB, in which inherited *COL7A1* mutations lead to Col VII deficiency or dysfunction, EBA represents an immune-mediated model of Col VII-related DEJ damage. Anti-Col VII antibodies target anchoring fibril components within the basement membrane zone and impair stable adhesion between the epidermis and dermis. Clinically, EBA can present with mechanobullous or inflammatory phenotypes, reflecting the heterogeneity of Col VII-directed autoimmune injury.

Therapeutic research in EBA mainly focuses on suppressing pathogenic anti-Col VII immune responses and controlling secondary inflammatory tissue injury. Conventional treatments include systemic corticosteroids, dapsone, colchicine, and other immunomodulatory agents, while severe or refractory cases may require immunosuppressants, intravenous immunoglobulin, rituximab, plasmapheresis, immunoadsorption, or extracorporeal photochemotherapy (62). However, treatment responses remain variable because EBA is rare and clinically heterogeneous, and high-quality controlled evidence is still limited. From the perspective of Col VII-related therapeutic research, EBA demonstrates that Col VII is not only essential for anchoring fibril formation and DEJ stability, but can also serve as a pathogenic autoantigen in autoimmune blistering disease. Serological assays based on recombinant NC1 and NC2 domains have been used to support EBA diagnosis and monitor anti-Col VII antibody titers, further confirming the central role of Col VII in disease mechanism and therapeutic evaluation.

### 3.3 Col VII in broader skin repair contexts

Evidence regarding Col VII in broader skin repair contexts remains relatively limited and is derived mainly from studies of DEJ basement membrane reconstruction following wounding, aged and photodamaged skin, and tissue-engineered skin and skin-equivalent models. The teams led by Rittié and Fisher found in a human partial-thickness wound model that the DEJ basement membrane structure in young adult skin can recover within approximately 4 weeks after wounding, with type IV collagen and Col VII gradually depositing after wound closure; in contrast, the DEJ basement membrane in aged skin shows insufficient recovery, suggesting that basement membrane zone reconstruction can serve as an important dimension for evaluating wound repair quality (63). In studies on photoaging, the DEJ basement membrane of sun-exposed skin may exhibit multilayering, localized breaks, and structural disorganization, suggesting that damage to the basement membrane zone may contribute to photoaging-related changes in skin structure (3). Furthermore, UVA and its associated inflammatory microenvironment can influence the transcriptional levels of *COL7A1* in keratinocytes, and the regulation of *COL7A1* expression by TNF-α and IL-1β exhibits cell-type-specific differences, suggesting that inflammatory states may alter the maintenance processes of Col VII-associated junctional structures (64). In studies of engineered skin and skin analogs, Col VII is primarily used to evaluate DEJ maturation, basement membrane formation, and the stability of epidermal-dermal junctions. Mimicking the natural DEJ helps enhance adhesion and physical integration between the epidermis and dermis; fibroblasts and the dermal microenvironment also promote the deposition of basement membrane components, including type IV collagen and Col VII (14, 65). Therefore, Col VII may serve as a functional indicator for evaluating basement membrane zone restoration, the stability of newly formed epithelium, and the maturation of engineered skin. Whether rhCol VII can be further developed as a topical repair material or a functional component of engineered skin remains to be determined through additional preclinical and clinical studies.

## 4 Potential applications of rhCol VII in skin repair and regeneration

### 4.1 Application basis and positioning of rhCol VII

**Figure 2:**
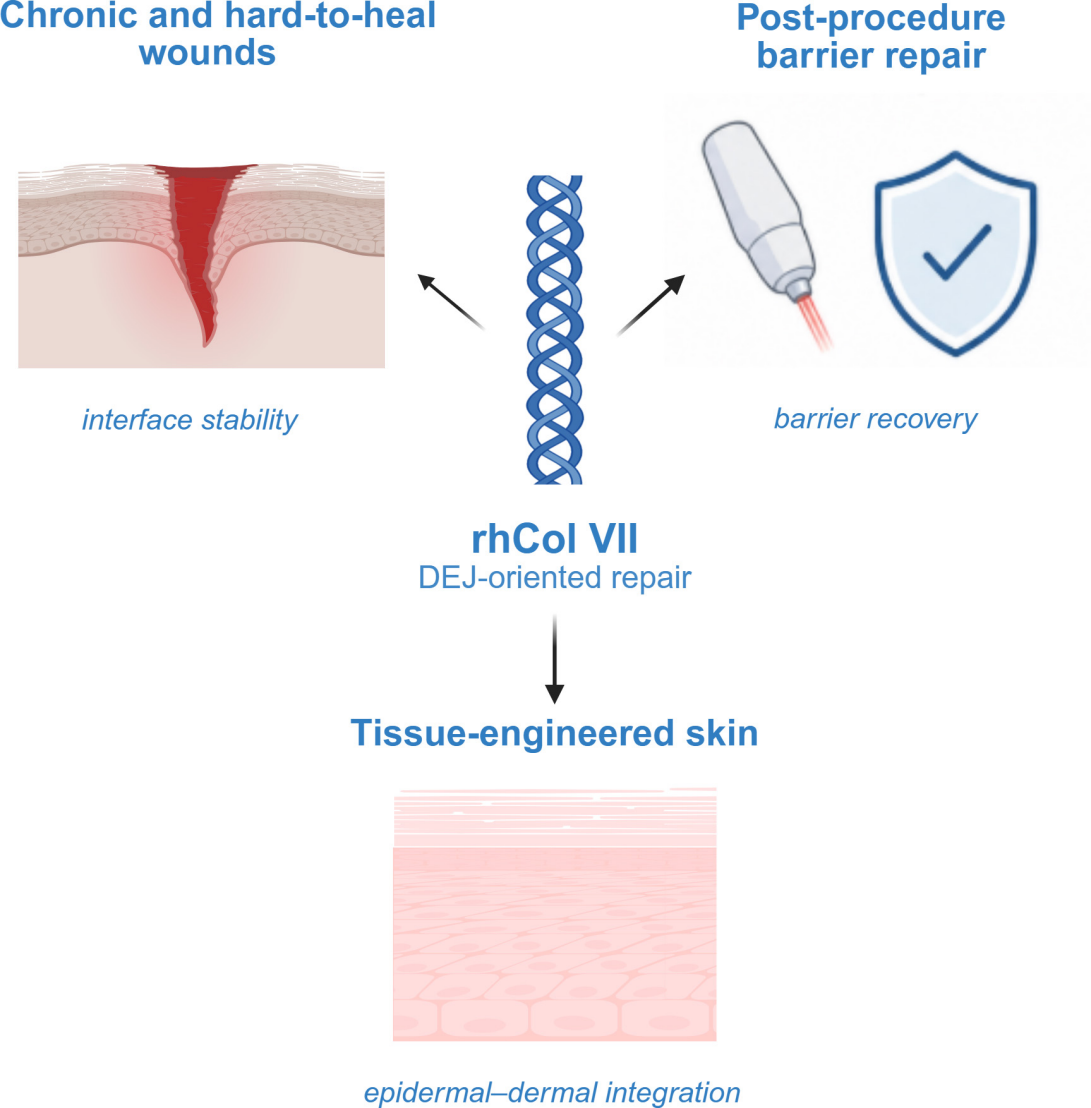
*Conceptual overview of the potential applications of rhCol VII in skin repair and regeneration*. rhCol VII is proposed as a DEJ-oriented biomaterial and may contribute to interface stability in chronic and hard-to-heal wounds, barrier recovery after skin procedures, and epidermal–dermal integration in tissue-engineered skin. Created with BioRender.com.

As a functional recombinant collagen designed for DEJ-oriented repair, rhCol VII is valuable because it links the general biocompatibility of collagen-based materials with the interface-repair potential of native Col VII. Recently, Lan et al. (66) constructed an rhCol VII with triple-helical structural features and showed favorable biosafety and repair potential *in vitro* and in an acute skin wound model, providing early support for rhCol VII material development. Whether different forms of rhCol VII can achieve stable tissue localization, retain functional domains, and sustain biological activity will depend on specific structural designs and experimental evidence. For this reason, rhCol VII should be regarded as a functional recombinant collagen distinct from conventional type I and type III collagen materials, and as a candidate for more precise skin repair material development. The conceptual framework of rhCol VII as a DEJ-oriented biomaterial and its potential applications in skin repair and regeneration are illustrated in Figure 2. Building on this framework, the following sections discuss its potential roles in chronic and hard-to-heal wounds, post-procedure barrier repair, and tissue-engineered skin. Table 1 provides a comparative overview of **t**he rationale for each application, evaluation indicators, potential advantages over type I and type III collagen, and current limitations of the available evidence.

**Table 1:** Potential applications of rhCol VII in skin repair and regeneration.

Application scenario	Main repair challenge	Proposed role of rhCol VII	Comparative advantage over type I/III collagen	Suggested evaluation indicators	Current evidence and limitations
Chronic and hard-to-heal wounds	Persistent inflammation, abnormal protease activity, impaired ECM remodeling, and unstable post-closure repair	May support DEJ reconstruction, improve basement membrane continuity, and enhance post-closure tissue stability	More directly targets basement membrane continuity and post-closure epidermal–dermal anchoring, beyond dermal ECM support	Col VII deposition; DEJ integrity; basement membrane continuity; transepidermal water loss recovery; recurrence rate	Early experimental evidence suggests repair potential, but clinical efficacy in diabetic foot ulcers, pressure injuries, and venous ulcers remains to be demonstrated
Post-aesthetic-procedure barrier damage	Laser, microneedling, and related procedures can cause barrier disruption, erythema, sensitivity, increased transepidermal water loss, and local inflammation	May provide a DEJ-oriented repair strategy beyond moisturizing, soothing, and epidermal regeneration	May provide DEJ-oriented repair cues after barrier disruption, beyond hydration, soothing, and dermal matrix support	transepidermal water loss recovery; erythema resolution; barrier recovery time; post-procedure sensitivity; Col VII deposition; DEJ integrity	Current support is mainly indirect; comparative studies are needed to clarify whether rhCol VII provides added benefit over conventional post-procedure repair products
Tissue-engineered skin	Immature DEJ-like interface and insufficient epidermal–dermal adhesion may limit graft stability and integration	May serve as a functional interface component to promote DEJ-like structure formation and improve graft attachment	Provides anchoring fibril-related interface cues to support epidermal–dermal adhesion, DEJ-like structure formation, and graft integration.	Epidermal–dermal adhesion; Col VII deposition; DEJ continuity; mechanical stability; graft integration	Supported by tissue-engineering rationale, but further preclinical validation is needed to confirm its contribution to engineered skin stability

### 4.2 Repair of chronic and hard-to-heal wounds

Chronic and hard-to-heal wounds may represent a promising application area for rhCol VII in skin repair. These wounds often involve persistent inflammation, abnormal protease activity, and impaired extracellular matrix remodeling (67, 68). Recent clinical research indicates that visible closure is not equivalent to functional closure. Closed wounds may still have barrier defects, and higher transepidermal water loss is associated with risk of chronic wound recurrence (69, 70). Accordingly, assessment of chronic wound repair should extend beyond closure rate and healing time to include post-closure functional stability and recurrence risk.

From a mechanistic perspective, Col VII is closely associated with keratinocyte migration, basement membrane remodeling, and wound healing processes. Therefore, in the healing of chronic and hard-to-heal wounds, Col VII serves as a key molecular marker reflecting the reconstruction of the DEJ and the stability of the skin following closure (71). Recently, rhCol VII with a triple-helix structure, developed by Lan et al., has demonstrated certain wound-healing effects, promoting wound closure and improving the structure of newly formed tissue, the fibroblast response, and extracellular matrix regeneration; simultaneously, histological results showed increased collagen deposition and matrix maturation following rhCol VII treatment, suggesting that it has certain potential for skin repair as a functional collagen material (66). However, the clinical efficacy of rhCol VII in areas such as diabetic foot, pressure injuries, and venous ulcers cannot yet be directly demonstrated. Therefore, future studies should focus on the stability and local retention capacity of rhCol VII in the microenvironment of complex wounds to determine whether it can facilitate Col VII deposition at the DEJ and improve basement membrane continuity. These studies should also incorporate indicators such as barrier restoration and recurrence rates to assess whether rhCol VII can further improve healing quality beyond that achieved with traditional collagen dressings.

### 4.3 Post-procedure barrier repair after aesthetic treatments

Aesthetic procedures such as laser resurfacing, microneedling, and superficial ablative treatments are designed to induce controlled skin injury and subsequent tissue remodeling, thereby improving scars, photoaging-related changes, skin texture, or other cosmetic concerns. However, these procedures also transiently disrupt the stratum corneum and epidermal barrier, leading to increased transepidermal water loss, local inflammation, erythema, dryness, and skin sensitivity (72). Current post-procedure repair products mainly focus on moisturization, soothing, anti-inflammatory effects, and epidermal barrier recovery (73, 74). In this context, rhCol VII may serve as a potential functional molecule targeting DEJ repair, providing a new perspective for post-procedure barrier restoration.

Full-length Col VII and most rhCol VII-derived functional fragments are large biomolecules. They are unlikely to passively diffuse through an intact stratum corneum and reach deeper skin structures. Therefore, the potential topical use of rhCol VII in aesthetic medicine should not be considered a conventional transdermal delivery approach for intact skin. Instead, its feasibility is more closely related to the transient barrier-disrupted window created by procedures such as fractional laser treatment, microneedling, or superficial ablation. These interventions, which are performed for their own therapeutic purposes, temporarily generate microchannels or exposed repair interfaces. This post-procedure window may allow rhCol VII to maintain local contact and retention near the injured epidermis and DEJ-associated repair region, thereby providing potential support for neoepidermal adhesion, basement membrane zone remodeling, and DEJ structural stability.

Accordingly, the role of rhCol VII in post-procedure repair should be considered in terms of structural repair rather than simple symptomatic relief. As a molecule closely associated with anchoring fibrils at the DEJ, rhCol VII is particularly relevant to neoepidermal attachment, basement membrane zone continuity, and DEJ stability. Its functional evaluation in this setting should therefore focus not only on conventional barrier-related outcomes, but also on local protein distribution, basement membrane zone integrity, DEJ organization, and the stability of newly repaired skin.

### 4.4 Tissue-engineered skin

Tissue-engineered skin is another area in which rhCol VII deserves further study. One of the major challenges in this field is rebuilding a DEJ that provides both mechanical adhesion and signaling regulation. Mimicking the natural DEJ can strengthen epidermal-dermal adhesion in engineered skin and improve graft integration (14). Introducing rhCol VII into skin substitutes could help form a more stable interface and support more reliable attachment to the underlying dermal-like matrix. In skin equivalents, collagen-based scaffolds, and keratinocyte-fibroblast co-culture systems, rhCol VII may also serve as a functional component for evaluating and optimizing DEJ reconstruction. Pre-incorporation of rhCol VII into the interface region could give artificial skin an initial DEJ-like functional basis before transplantation, reducing complete dependence on host tissue to rebuild the DEJ after grafting and potentially improving early attachment and long-term integration.

## 5 Material development and translational evaluation of rhCol VII-based biomaterials

### 5.1 Recombinant production and structural challenges of full-length rhCol VII

The development of recombinant humanized type III collagen (rhCol III) has provided a relatively mature model for the industrialization of recombinant humanized collagen, particularly in terms of sequence design, recombinant expression, structural characterization, biocompatibility evaluation, and skin-related applications, with available preclinical and clinical studies generally indicating a favorable safety profile. In China, Jinbo Biotech pioneered this field with its rhCol III freeze-dried fibers, which became the first Class III implantable medical device based on recombinant human-derived collagen technology to obtain marketing approval from the China National Medical Products Administration (NMPA). Currently, the application evaluation of rhCol III primarily focuses on skin photoaging repair, dermal fillers, and tissue engineering, all of which have demonstrated good *in vivo* biosafety, anti-aging and repair potential, and clinical tolerability; no serious adverse events have been reported in related studies (75–77). Therefore, rhCol III provides an important reference for the development of recombinant humanized collagen materials.

Compared with rhCol III, full-length rhCol VII is much larger and structurally more complex, as each α1(VII) chain is approximately 300 kDa and three chains assemble into a triple-helical trimer of approximately 900 kDa. Structurally, Col VII contains a large N-terminal NC1 domain, a central collagenous triple-helical domain, and a smaller C-terminal NC2 domain, all of which are involved in basement membrane binding, molecular dimerization, and anchoring fibril assembly (78). Therefore, producing functional full-length rhCol VII requires not only expression of a large collagen chain, but also correct chain association, triple-helical folding, NC2-mediated antiparallel dimerization, secretion, and preservation of functional domains required for DEJ localization. In addition, collagen-specific post-translational modifications, particularly prolyl 4-hydroxylation of proline residues in Gly-X-Y repeats, are essential for maintaining triple-helix stability at physiological temperature (79, 80). Thus, selecting an expression system that supports collagen-specific hydroxylation, correct folding, secretion, and structural maturation is critical for producing biologically active full-length rhCol VII. These structural and biosynthetic requirements may substantially increase the complexity of large-scale production, purification, batch-to-batch consistency control, quality assessment, and cost management, thereby posing considerable challenges to the industrial development of full-length rhCol VII.

Mammalian expression systems, such as HEK293 and CHO cells, are generally advantageous for producing complex human proteins because they support eukaryotic folding, secretion, and more human-like post-translational processing (81, 82). For Col VII specifically, full-length rhCol VII has been expressed in stably transfected human 293 cells and purified from culture medium, and CHO-derived rhCol VII has also been reported to form a correctly folded, disulfide-bonded, helical trimer with biological activity (20, 83). These findings suggest that mammalian systems are more favorable when structural fidelity and functional maturation of full-length rhCol VII are required. However, mammalian cell-based production is generally associated with higher culture costs, more complex cell-line development, and greater challenges in yield improvement and large-scale manufacturing.

Yeast systems, such as Pichia pastoris, offer advantages in rapid growth, high-density fermentation, scalability, and cost efficiency. However, yeast cells do not naturally provide sufficient collagen-specific prolyl 4-hydroxylase activity, and co-expression of prolyl 4-hydroxylase is often required to obtain hydroxylated recombinant collagen with a stable triple-helical structure (84, 85). Thus, yeast systems may be more practical for collagen fragments, collagen-like domains, or engineered functional modules, whereas full-length rhCol VII production places higher demands on hydroxylation, secretion, folding, purification, and batch-to-batch structural consistency.

### 5.2 Immunogenicity and autoimmune safety

Col VII is the major autoantigen in EBA, an acquired autoimmune subepidermal blistering disease mediated by tissue-bound and circulating immunoglobulin G (IgG) autoantibodies against Col VII at the basement membrane zone (60). Because rhCol VII is an exogenous humanized collagen derived from the same target molecule, its translational evaluation should consider not only general biocompatibility but also the potential risk of inducing or amplifying anti-Col VII autoimmune responses. This concern is particularly relevant in therapeutic settings that may require repeated administration or prolonged local exposure.

Nevertheless, available preclinical studies of rhCol VII or recombinant Col VII provide a generally reassuring immune safety profile rather than evidence of a strong tendency to trigger EBA-like autoimmunity. In Col VII-null DEB mice, intradermally injected rhCol VII incorporated into the basement membrane zone, formed anchoring fibrils, improved the DEB phenotype, and prolonged survival (44). Although circulating anti-human Col VII antibodies were detected after repeated exposure, these antibodies did not bind to the basement membrane zone, did not induce complement component 3 deposition, and did not prevent subsequent incorporation of newly injected Col VII (44). In another study, intravenously administered CHO-derived rhCol VII incorporated into the DEJ, restored anchoring fibrils, improved dermal–epidermal adherence, and increased survival in Col VII-deficient RDEB mice, with no detectable circulating or tissue-bound anti-Col VII antibodies (83). Repeated intravenous administration of recombinant Col VII was also well tolerated in adult RDEB mice and dogs, further supporting the feasibility of repeated systemic exposure in preclinical models (86). These findings suggest that anti-Col VII antibody formation, when observed, does not necessarily translate into pathogenic EBA-like autoimmune disease, thereby providing an important safety basis for the continued development of rhCol VII.

From the broader perspective of recombinant humanized collagen development, human-sequence design and controllable recombinant production may reduce safety concerns associated with animal-derived collagen. However, long-term biosafety should still be interpreted with attention to anti-Col VII antibody formation, immune deposition at the DEJ, delayed blistering, and other autoimmune-related changes during repeated or prolonged use.

### 5.3 Delivery strategies and functional evaluation endpoints

From the perspectives of formulation and clinical translation, rhCol VII could be explored in various delivery methods, such as hydrogels, dressings, topical application following microneedling, or combined use with cells or scaffolds; however, the risks associated with these different scenarios vary. For chronic wounds, its stability and retention time in inflammatory and protease-rich environments should be demonstrated; for postoperative barrier damage, its added benefits compared to conventional moisturizing and anti-inflammatory products must be demonstrated; and for tissue-engineered skin, its ability to improve DEJ continuity and mechanical stability of the graft should be demonstrated. Therefore, future studies should conduct a combined evaluation of wound closure rates alongside indicators such as Col VII deposition, basement membrane continuity, DEJ integrity, transepidermal water loss, recurrence or scarring, and local immune responses to determine whether rhCol VII truly achieves stable interface reconstruction.

## 6 Conclusion and outlook

Col VII is a central structural component of anchoring fibrils at the DEJ, where it contributes to stable adhesion between the epidermis and dermis. In addition to its mechanical role, Col VII is closely associated with re-epithelialization, basement membrane reconstruction, and the stabilization of newly formed skin after injury. Research on Col VII restoration in DEB has provided important evidence regarding the biological and therapeutic relevance of this protein. Meanwhile, studies of wound healing, aged and photodamaged skin, and tissue-engineered skin have further highlighted Col VII as a potential indicator of DEJ reconstruction and tissue maturation. Collectively, these findings support further investigation of rhCol VII as a functional biomaterial oriented toward DEJ repair.

Compared with type I and type III collagen materials, which are primarily used to support dermal matrix regeneration, the potential value of rhCol VII lies in its functional association with junctional repair and epidermal–dermal interface stability. Based on this positioning, chronic and hard-to-heal wounds, post-procedure barrier repair, and tissue-engineered skin may represent three potential application directions. However, the available evidence remains limited and is derived largely from preclinical studies or indirect observations of native Col VII. Accordingly, the evaluation of rhCol VII should extend beyond wound closure and short-term barrier recovery to include Col VII deposition at the basement membrane zone, basement membrane continuity, DEJ integrity, transepidermal water loss, and the mechanical stability of repaired skin. Application-specific outcomes, such as wound recurrence and graft integration, should also be considered where appropriate.

From a translational perspective, recombinant human-sequence design provides a potential route toward improved source control and standardized manufacturing. Nevertheless, the production of functionally active rhCol VII requires appropriate triple-helical folding, collagen-specific post-translational modifications, preservation of functional domains, and reproducible structural properties. Further development will also depend on effective delivery and local retention at the repair interface, stability in inflammatory and protease-rich environments, batch-to-batch consistency, and long-term immune safety during repeated or prolonged use. Future studies should therefore link structural and quality attributes of rhCol VII with application-specific functional outcomes and compare its performance with established collagen materials and current repair strategies. With further structural, preclinical, and clinical validation, rhCol VII may provide a distinct DEJ-oriented approach for skin repair and regenerative medicine.
